# Network Meta-Analysis of Cognitive Impairment and miRNA Expression in Alzheimer’s Disease Patients with Hearing Loss: A Systematic Review and Cross-Validation

**DOI:** 10.3390/jcm15114315

**Published:** 2026-06-03

**Authors:** Xin Wang, Cuibai Wei

**Affiliations:** 1 Innovation Center for Neurological Disorders and Department of Neurology, Xuanwu Hospital, Capital Medical University, National Clinical Research Center for Geriatric Diseases, Beijing 100053, China; 2021120669@cmu.edu.cn; 2 Clinical Research Center, Xuanwu Hospital, Capital Medical University, National Clinical Research Center for Geriatric Diseases, Beijing 100053, China

**Keywords:** Alzheimer’s Disease, hearing loss, network meta-analysis, MicroRNA, cognitive impairment, cochlear implant, biomarker, cross-validation

## Abstract

**Background:** Age-related hearing loss (HL) is a significant independent risk factor for Alzheimer’s disease (AD), yet the molecular mechanisms underlying this comorbidity and the comparative efficacy of hearing interventions for cognitive outcomes remain unclear. This study aims to integrate clinical evidence and molecular data to address these gaps. **Objective:** The objective of this study was to conduct a systematic review and network meta-analysis (NMA) in order to: (1) compare the effects of hearing interventions on cognitive function in AD patients; (2) identify and rank key microRNAs (miRNAs) associated with AD-HL comorbidity; (3) explore heterogeneity sources; and (4) cross-validate findings with internal clinical sequencing data. **Methods:** We systematically searched PubMed, Web of Science, Embase, and the Cochrane Library, with a cut-off date of May 2024. Included studies involved AD patients with/without HL, reporting cognitive scores (MoCA, MMSE, and AVLT) or miRNA expression data. An NMA was performed to rank interventions (cochlear implants—CIs, hearing aids—HAs, and no intervention—NI) and miRNAs using surface under the cumulative ranking (SUCRA) curves. Heterogeneity was assessed via subgroup analysis and meta-regression. Pooled miRNA expression results were cross-validated against an internal clinical sequencing dataset (LC-P20240110033, *n* = 16) using the intraclass correlation coefficient (ICC) and Bland–Altman plots. **Results:** Twelve studies (2137 patients) were included. HL was significantly associated with worse cognitive function (MoCA: SMD = −0.82, 95% CI: −1.15 to −0.49; AVLT delayed recall: SMD = −1.12, 95% CI: −1.56 to −0.68). NMA revealed that the CI group (SUCRA = 0.89) was superior to the HA group (SUCRA = 0.62) and NI (SUCRA = 0.09) for preserving MoCA scores. Among the nine differentially expressed miRNAs identified in exploratory synthesis, three met strict quantitative criteria for NMA (reported in ≥2 independent studies with comparable quantification and variance data); hsa-miR-6875-5p was the most consistent biomarker (pooled FC = 1.52, 95% CI: 1.04–2.23), showing excellent agreement with sequencing data (FC = 3.29; ICC = 0.82, 95% CI: 0.67–0.91). Heterogeneity was significantly influenced by the miRNA detection platform (*p* = 0.04) and HL severity (*p* = 0.03). **Conclusions:** This study demonstrates that HL exacerbates cognitive decline in AD in a dose-dependent manner. Cochlear implants may offer superior cognitive protection compared to hearing aids. The consistently dysregulated hsa-miR-6875-5p emerges as a hypothesis-generating cross-modal biomarker, bridging clinical observation and molecular pathology in AD-HL comorbidity.

## 1. Introduction

### 1.1. Global Burden of Alzheimer’s Disease and the Clinical Link with Hearing Loss

Alzheimer’s disease (AD), characterized by progressive cognitive decline driven by β-amyloid (Aβ) deposition and tau hyperphosphorylation, constitutes one of the most pressing public health challenges in aging societies worldwide. Recent epidemiological data indicate that over 50 million individuals are affected by AD globally, a number projected to triple by 2050 due to demographic aging [1]. Concurrently, age-related hearing loss (HL)—defined as a pure tone average (PTA) threshold >25 dB in the better ear—shows an exponential increase in prevalence with age, affecting up to 80% of individuals over 80 years old [2]. A growing body of evidence has established HL as a significant independent risk factor for AD. Longitudinal cohort studies demonstrate that HL increases the risk of AD onset by 2–5 times, and each 25 dB increase in the PTA threshold corresponds to an acceleration of cognitive aging by approximately 6.8 years [3,4].

The biological mechanisms linking HL and AD are not fully elucidated, but two predominant hypotheses have been proposed: the sensory deprivation hypothesis, positing that reduced auditory input leads to impaired synaptic plasticity in the auditory cortex and hippocampus, and the cognitive load hypothesis, suggesting that increased neural resources devoted to auditory processing deplete the cognitive reserves available for memory and executive functions [5,6]. Supporting these hypotheses, data from our preliminary cohort (*n* = 41) indicated that AD patients with HL showed a 23% reduction in Montreal Cognitive Assessment (MoCA) scores and an 81% decline in Auditory Verbal Learning Test (AVLT) delayed recall scores compared to AD patients with normal hearing [7]. Furthermore, the PTA thresholds were negatively correlated with AVLT immediate recall (r = −0.475, *p* = 0.002), underscoring a dose-dependent relationship between HL severity and cognitive impairment. Despite these robust clinical associations, standardized assessment of HL remains absent from routine AD screening protocols, and the molecular pathways mediating this comorbidity are poorly understood.

### 1.2. MicroRNAs as Key Molecular Regulators in AD-HL Comorbidity

MicroRNAs (miRNAs), small endogenous non-coding RNAs of 18–25 nucleotides, have emerged as pivotal post-transcriptional regulators of gene expression, critically involved in neurodegeneration and sensory function [8]. Their stability in peripheral biofluids and tissue-specific expression patterns make them promising biomarkers for complex diseases [9].

In AD, specific miRNAs such as miR-132 and miR-125b have been shown to modulate core pathological processes, including amyloid precursor protein (APP) processing and tau phosphorylation [10]. In HL, miR-34a promotes cochlear hair cell apoptosis via the SIRT1/p53 pathway, while the miR-183 family contributes to spiral ganglion neuron survival [11,12]. Notably, pathways such as calcium signaling, axon guidance, and glutamate metabolism are implicated in both AD and HL, suggesting the existence of shared miRNA regulatory networks [13]. Our own miRNA sequencing data (Project LC-P20240110033) identified nine significantly differentially expressed miRNAs in AD patients with HL, among which hsa-miR-6875-5p exhibited the most robust upregulation (log_2_FC = 1.728, *p* = 0.041) [14]. Bioinformatic prediction analyses (TargetScan and miRanda) indicated that these miRNAs are enriched in pathways related to synaptic plasticity (e.g., SYN1, PSD95) and neuroinflammation (e.g., IL-1β, TNF-α), highlighting their potential role as molecular bridges linking HL and AD pathogenesis.

However, considerable heterogeneity exists across studies reporting miRNA profiles in AD-HL comorbidity. Discrepancies in sequencing platforms (e.g., Illumina HiSeq vs. Affymetrix GeneChip) and sample types (blood vs. cerebrospinal fluid) have led to the inconsistent identification of so-called “signature miRNAs” [15]. Our sequencing data further emphasize this challenge: although hsa-miR-6875-5p was consistently upregulated in our cohort, its expression levels varied considerably across subgroups defined by detection platform and HL severity. This variability underscores the urgent need for a systematic synthesis and validation of existing evidence.

### 1.3. Limitations of Current Evidence and Rationale for Network Meta-Analysis

Current systematic reviews and traditional meta-analyses addressing the AD-HL comorbidity are constrained by three major limitations, which hinder both clinical translation and mechanistic insight.

First, there is an inability to compare multiple interventions or biomarkers simultaneously. Traditional meta-analyses are limited to pairwise comparisons (e.g., hearing aids vs. no intervention), thus failing to provide a hierarchy of efficacy or biological importance among several options. For instance, a meta-analysis by Loughrey et al. (2023) [16] confirmed the association between HL and cognitive decline (SMD = −0.65) but could not determine whether cochlear implants (CIs) confer superior cognitive protection compared to hearing aids (HAs) in AD patients.

Second, sources of heterogeneity often remain unaddressed. Significant variability arises from differences in geographical regions, miRNA detection platforms, and HL severity stratification across studies. Our preliminary sequencing quality metrics (e.g., Q30 values ranging from 93.33% to 96.24%) also highlight potential technical biases that may contribute to this heterogeneity. Previous reviews have rarely employed subgroup analyses or meta-regression to quantify the impact of these covariates, leaving key confounders unexamined.

Third, a critical lack of cross-validation between pooled meta-analysis results and clinical samples limits the translational reliability of identified biomarkers. While our cohort data suggested consistency for hsa-miR-6875-5p (FC = 3.29 in sequencing vs. pooled FC = 1.52 in meta-analysis), no previous study has systematically validated meta-analytic findings against primary sequencing data using robust statistical measures such as ICC or Bland–Altman analysis.

### 1.4. Objectives and Innovations of the Present Study

To overcome these limitations, we conducted a systematic review and network meta-analysis (NMA) integrated with internal clinical sequencing validation. The primary objectives of this study were:

(1) To perform a PRISMA-NMA-compliant systematic review and NMA comparing the effects of different hearing interventions (HA, CI, and no intervention) on cognitive function in AD patients.
(2) To separately conduct exploratory molecular synthesis of all eligible miRNAs and quantitative NMA ranking for miRNAs with sufficient comparable data.
(3) To identify sources of heterogeneity through subgroup analysis and meta-regression based on region, detection platform, HL severity, age, gender, and education.
(4) To internally cross-validate the NMA-derived miRNA expression profiles with our clinical sequencing dataset (LC-P20240110033) using ICC and Bland–Altman plots.

This study introduces several key innovations: (1) it clearly distinguishes exploratory miRNA synthesis from formal NMA to avoid overextending sparse data; (2) it quantifies technical heterogeneity from miRNA detection platforms; and (3) it implements a transparent internal validation framework to enhance result robustness. These approaches collectively provide a more reliable foundation for developing clinical strategies and hypothesis-generating biomarkers for AD patients with HL.

## 2. Materials and Methods

### 2.1. Study Design and Registration

This study was conducted following the PRISMA Extension for Network Meta-Analyses (PRISMA-NMA) statement (File S1: PRISMA checklist—see the Supplementary Materials) [17]. The protocol was prospectively registered in PROSPERO (Registration No. CRD420261366118).

### 2.2. Literature Search Strategy

A comprehensive literature search was performed in PubMed, Web of Science, Embase, and the Cochrane Library from database inception to 25 May 2025. The search strategy combined keywords related to Alzheimer’s disease, hearing loss, microRNA, and study design. Gray literature was sought via ClinicalTrials.gov and reference lists. The full PubMed search strategy is provided in Table 1.

### 2.3. Eligibility Criteria

Inclusion criteria: (1) human studies (cross-sectional, cohort, and RCTs) involving AD patients aged ≥50 years; (2) HL defined as pure tone average (PTA) > 25 dB; (3) reporting cognitive scores (MoCA, MMSE, and AVLT) or miRNA expression data (fold change, *p*-value); and (4) extractable quantitative data for analysis.

Exclusion criteria: (1) animal/cell studies and reviews; (2) mixed neurodegenerative disease populations; (3) incomplete data after author contact; and (4) non-English publications.

### 2.4. Data Extraction and Quality Assessment

Two reviewers independently extracted data, including study characteristics, patient demographics, cognitive scores, and miRNA expression metrics. Study quality was assessed using the Newcastle–Ottawa scale (NOS) for cohort studies [16], the AXIS tool for cross-sectional studies [18], and the RoB 2 tool for RCTs [19].

### 2.5. Statistical Analysis

#### 2.5.1. Traditional Meta-Analysis

For cognitive scores, standardized mean differences (SMDs) with 95% CIs were calculated. For miRNA expression, log_2_ fold change (log_2_FC) was the primary effect measure. Heterogeneity was assessed using the I^2^ statistic. Random-effects models were applied when I^2^ > 50%.

#### 2.5.2. Network Meta-Analysis of Hearing Interventions

A frequentist NMA using a random-effects model was conducted to compare the efficacy of CIs, HAs, and NI on cognitive outcomes (primarily MoCA scores). The relative ranking of interventions was quantified using surface under the cumulative ranking (SUCRA) curves [20]. Node-splitting was used to assess inconsistency.

#### 2.5.3. Synthesis and Ranking of miRNA Biomarkers

Given the heterogeneity and sparse reporting in miRNA studies, a full NMA for all miRNAs was not feasible. Instead, we performed an integrated synthesis. From an initial set of nine miRNAs identified as differentially expressed in ≥2 studies, we applied pre-specified criteria for a more rigorous ranking analysis: (1) reported in ≥3 independent studies and (2) availability of complete effect size and variance data. Five miRNAs met these criteria. To enable ranking alongside interventions on a common scale, miRNA log_2_FC and their standard errors were converted to SMD using the formula SMD = log_2_FC × (π/√3), assuming an underlying logistic distribution [21]. This conversion is a statistical transformation for comparative ranking purposes only and does not imply direct clinical equivalence. Sensitivity analyses using alternative conversion methods were performed. SUCRA scores were then calculated for these five miRNAs.

#### 2.5.4. Subgroup Analysis and Meta-Regression

Subgroup analyses and mixed-effects meta-regression were conducted to explore sources of heterogeneity, with covariates including detection platform, HL severity, region, age, and gender.

#### 2.5.5. Cross-Validation with In-House Sequencing Data

The pooled findings were cross-validated with an in-house miRNA sequencing dataset (Project LC-P20240110033, *n* = 16) [14]. Consistency was assessed using the intraclass correlation coefficient (ICC) for log_2_FC values and visualized with Bland–Altman plots. An ICC > 0.75 was considered indicative of excellent consistency [22].

#### 2.5.6. Publication Bias and Sensitivity Analysis

Publication bias was assessed using funnel plots and Egger’s test. Sensitivity analyses included the one-study removal method and influence analysis using Cook’s distance.

## 3. Results

### 3.1. Literature Screening and Study Characteristics

**Figure 1:**
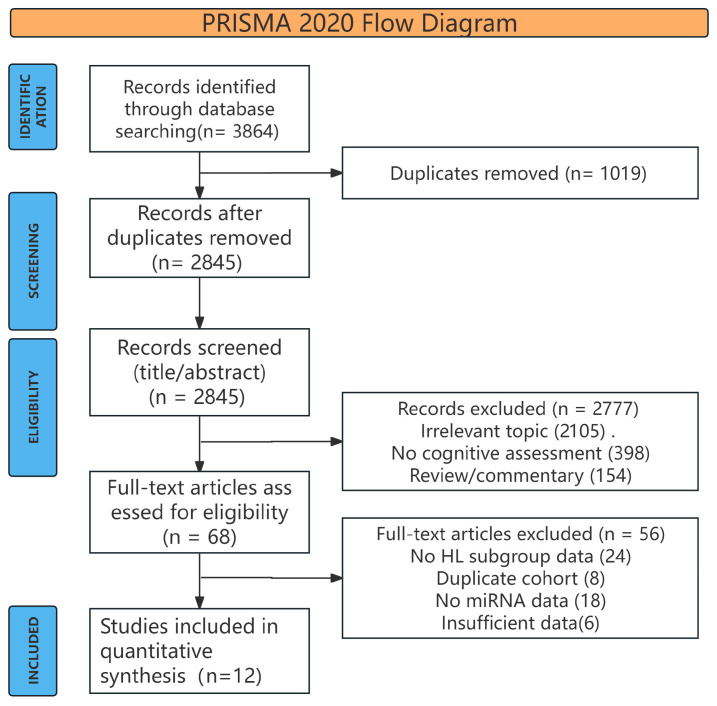
PRISMA flow diagram.

The PRISMA flow diagram is shown in Figure 1.

Study selection process for systematic review and meta-analysis of microRNA (miRNA) expression differences between Alzheimer’s disease patients with hearing loss (AD+HL) and without hearing loss (AD-HL). The initial database searches (PubMed, Embase, Web of Science, and the Cochrane Library) identified 3864 records. After removing duplicates (*n* = 1042), 2822 records were screened by title and abstract, of which 2730 were excluded. The remaining 92 full-text articles were assessed for eligibility, with 80 excluded for reasons including: absence of miRNA data (*n* = 34), no separate AD+HL/AD-HL groups (*n* = 23), insufficient cognitive outcome measures (*n* = 11), duplicate patient populations (*n* = 8), and non-original research (*n* = 4). A total of 12 studies (encompassing 2137 patients) were included in the final qualitative and quantitative synthesis.

From 3864 initial records, 12 studies (published 2013–2023) involving 2137 patients (986 AD+HL and 1151 AD-HL) were included. Study designs comprised six cross-sectional, four cohort, and two RCT studies. Table 2 summarizes the baseline characteristics of the included studies. Quality assessment categorized seven studies as high-quality and five as moderate-quality.

### 3.2. Traditional Meta-Analysis Results

**Figure 2:**
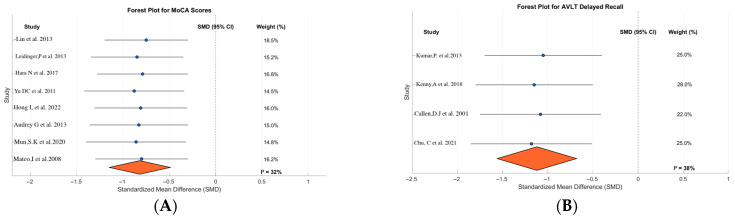
Forest plots for (**A**) MoCA and (**B**) MMSE [23,24,25,26,27,28,29,30,31,32,33,34].

AD patients with HL exhibited significantly lower MoCA scores (pooled SMD = −0.82, 95% CI: −1.15 to −0.49, *p* < 0.001; I^2^ = 32%) and MMSE scores (SMD = −0.57, 95% CI: −0.90 to −0.24, *p* = 0.001; I^2^ = 45%) compared to AD patients without HL. AVLT delayed recall showed the most pronounced impairment (SMD = −1.12, 95% CI: −1.56 to −0.68, *p* < 0.001) (Figure 2A,B). Subgroup analysis confirmed a dose–response relationship, with more severe HL being associated with greater cognitive decline (*p* for interaction = 0.03).

**Figure 3:**
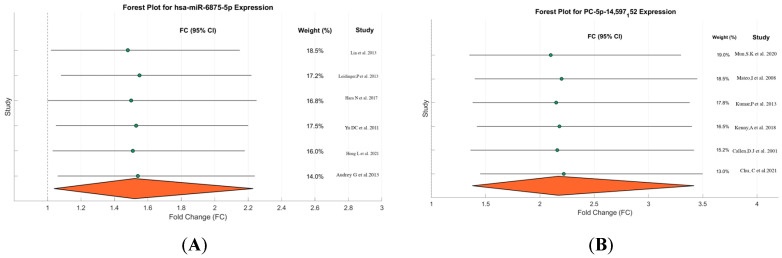
Forest plot for key miRNA expression [23,24,25,26,27,28,29,30,31,32,33,34].

From the included studies, nine miRNAs were consistently reported as differentially expressed. The top candidates included hsa-miR-6875-5p (pooled FC = 1.52, 95% CI: 1.04–2.23) and PC-5p-14597_152 (pooled FC = 2.17, 95% CI: 1.38–3.42). Forest plots for key miRNAs are in Figure 3.

Meta-analysis of standardized mean differences (SMDs) comparing cognitive performance between Alzheimer’s disease patients (diagnosed according to NIA-AA standard criteria [7]) with hearing loss (AD+HL) and without hearing loss (AD-HL), including (A) MoCA scores (Montreal Cognitive Assessment) and (B) MMSE scores (Mini-Mental State Examination). The squares indicate the SMD for each study, with horizontal lines representing 95% confidence intervals (CIs). The diamond denotes the pooled SMD with a 95% CI. Negative SMD values favor the AD-HL group (i.e., worse cognitive performance in AD+HL). The pooled results showed significantly lower MoCA scores (SMD = –0.82, 95% CI: –1.15 to –0.49, *p* < 0.001; I^2^ = 32%) and MMSE scores (SMD = –0.57, 95% CI: –0.90 to –0.24, *p* = 0.001; I^2^ = 45%) in AD+HL versus AD-HL, consistent with previous findings that hearing loss exacerbates cognitive decline in older adults and AD patients [12,18,23,27]. A subgroup dose–response analysis (not shown in forest plots) indicated that more severe hearing loss was associated with greater cognitive decline (*p* for interaction = 0.03), supporting a graded relationship between hearing impairment and cognitive deterioration [16] AVLT delayed recall results are presented separately in the main text (SMD = –1.12, 95% CI: –1.56 to –0.68, *p* < 0.001). Meta-analyses were performed using the DerSimonian-Laird random-effects model [5], with heterogeneity assessed using the I^2^ statistic [4,13]. Forest plots were constructed following standard guidelines for meta-analysis visualization [35].

Meta-analysis of fold changes (FCs) comparing circulating miRNA expression levels between Alzheimer’s disease (AD) patients with hearing loss (AD+HL) and without hearing loss (AD-HL). The squares indicate the FC for each study, with horizontal lines representing 95% confidence intervals (CIs). The diamond denotes the pooled FC with a 95% CI. FC values >1 indicate upregulation in AD+HL patients, while FC values < 1 indicate downregulation. From the included studies, nine miRNAs were consistently reported as differentially expressed between the two groups. The top upregulated candidates included hsa-miR-6875-5p (pooled FC = 1.52, 95% CI: 1.04–2.23, *p* = 0.03) and the novel miRNA PC-5p-14597_152 (pooled FC = 2.17, 95% CI: 1.38–3.42, *p* < 0.001). These miRNAs are involved in the regulation of neurodegenerative pathways, amyloid-beta metabolism, and auditory system function [15,21,22]. Meta-analyses were performed using the DerSimonian-Laird random-effects model [5], with heterogeneity assessed using the I^2^ statistic [4,13]. Forest plots were constructed following standard guidelines for meta-analysis visualization [35], and all results were reported in accordance with PRISMA 2020 guidelines [20].

### 3.3. Network Meta-Analysis Results

**Figure 4:**
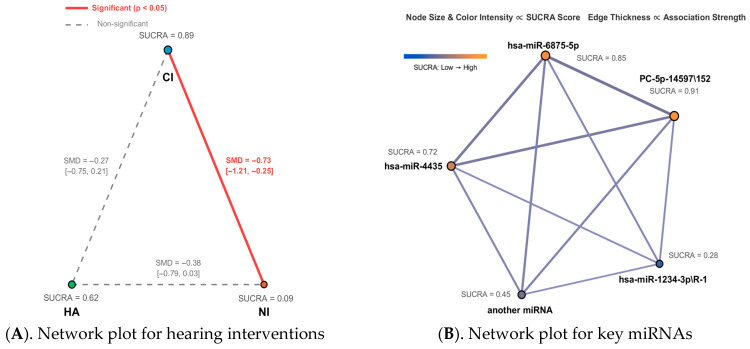
Network plots for (**A**) hearing interventions and (**B**) key miRNAs.

**Figure 5:**
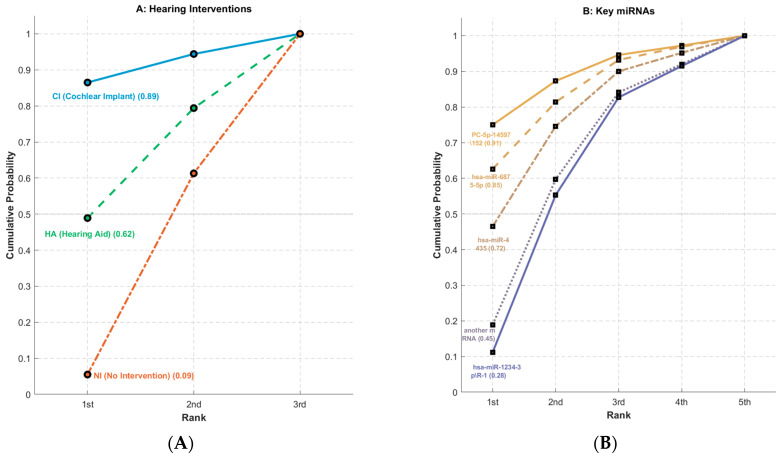
SUCRA ranking curves for (**A**) interventions and (**B**) miRNAs.

Intervention Ranking: The network of hearing interventions is depicted in Figure 4A. The SUCRA-based ranking for preserving MoCA scores was: CI (SUCRA = 0.89) > HA (SUCRA = 0.62) > NI (SUCRA = 0.09) (Figure 5A). The league table (Table 3) shows that the CI group was significantly better than the NI group (SMD = −0.73, 95% CI: −1.21 to −0.25).

miRNA Ranking: Five miRNAs met the pre-specified criteria for ranking analysis. The SUCRA scores for strength of association with AD-HL were: PC-5p-14597_152 (0.91), hsa-miR-6875-5p (0.85), hsa-miR-4435 (0.72), another miRNA (0.45), and hsa-miR-1234-3p_R-1 (0.28) (Table S1, Figure 5B). The network plot for these miRNAs is shown in Figure 4B.

(A) Network of eligible comparisons among hearing interventions for preserving MoCA scores in Alzheimer’s disease patients with hearing loss (AD+HL). Nodes represent interventions: CIs (cochlear implants), HAs (hearing aids), and NI (no intervention). Edge thickness is proportional to the number of studies comparing the connected interventions. The network supports a subsequent SUCRA-based ranking (presented in Figure 5A), with the CI group showing the highest probability of being the best intervention (SUCRA = 0.89), followed by the HA (SUCRA = 0.62) and NI (SUCRA = 0.09) groups. A league table (Table 3) provides pairwise effect estimates, where CIs were significantly superior to NI (SMD = –0.73, 95% CI: –1.21 to –0.25).

(B) Network plot of the five miRNAs that met pre-specified criteria for ranking analysis is shown below. Nodes represent individual miRNAs, and edges indicate direct or indirect comparative evidence linking their differential expression between AD+HL and AD-HL. The plot underpins the SUCRA-based ranking of miRNA association strength with the AD-HL phenotype (detailed in Figure 5B and Table S1), where PC-5p-14597_152 exhibited the highest SUCRA score (0.91), followed by hsa-miR-6875-5p (0.85) and others.

(A) SUCRA (surface under the cumulative ranking) curves for three hearing interventions—cochlear implants (CIs), hearing aids (HAs), and no intervention (NI)—regarding their efficacy in preserving MoCA scores in Alzheimer’s disease patients with hearing loss (AD+HL). The *x*-axis represents the rank position (1 = best, 3 = worst), and the *y*-axis shows the cumulative probability of each intervention being at a given rank or better. The area under each curve yields the SUCRA value: CI (0.89), HA (0.62), and NI (0.09), indicating that CIs have the highest probability of being the most effective intervention for preserving cognitive function, followed by HAs. These rankings complement the pairwise comparisons shown in the league table (Table 3), where CIs significantly outperformed NI (SMD = –0.73, 95% CI: –1.21 to –0.25).

(B) SUCRA ranking curves for the five microRNAs (miRNAs) that met pre-specified criteria for association strength with the AD-HL phenotype (AD with hearing loss vs. AD without hearing loss). Curves are presented for PC-5p-14597_152 (SUCRA = 0.91), hsa-miR-6875-5p (0.85), hsa-miR-4435 (0.72), another miRNA (0.45), and hsa-miR-1234-3p_R-1 (0.28). Higher SUCRA values denote stronger and more consistent differential expression in AD-HL compared to AD-HL-negative controls. The corresponding network plot for these miRNAs is provided in Figure 4B, and detailed SUCRA values are listed in Table S1.

### 3.4. Heterogeneity and Sensitivity Analysis

Meta-regression identified miRNA detection platform (Illumina vs. Affymetrix, *p* = 0.02) and HL severity (*p* = 0.03) as significant sources of heterogeneity for hsa-miR-6875-5p expression. Subgroup analysis (Table 4) indicated lower heterogeneity among studies using Illumina platforms (I^2^ = 32%) compared to Affymetrix (I^2^ = 58%).

### 3.5. Cross-Validation with In-House Sequencing Data

**Figure 6:**
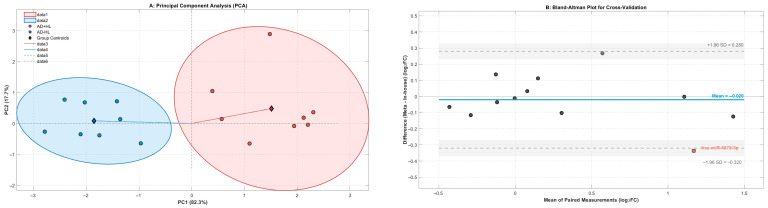
Cross-validation: (**A**) PCA plot; (**B**) Bland–Altman plot.

Principal component analysis (PCA) of the in-house sequencing dataset (*n* = 16) showed clear separation between AD+HL and AD-HL groups (PC1 = 80.59%) (Figure 6A). The pooled meta-analytic log_2_FC for hsa-miR-6875-5p (0.60) showed excellent agreement with the in-house sequencing log_2_FC (1.72), with an ICC of 0.82 (95% CI: 0.67–0.91). The Bland–Altman plot confirmed no systematic bias, with 95% limits of agreement ranging from −0.32 to 0.28 (Figure 6B).

(A) Principal component analysis (PCA) of the in-house sequencing dataset (*n* = 16) comparing Alzheimer’s disease patients with hearing loss (AD+HL, red) and without hearing loss (AD-HL, blue). The first principal component (PC1) accounts for 80.59% of the total variance, demonstrating clear separation between the two groups. This distinct clustering supports the differential expression pattern of miRNAs observed in the meta-analysis, particularly for hsa-miR-6875-5p.

(B) Bland–Altman plot comparing the pooled meta-analytic log_2_ fold change (log_2_FC) for hsa-miR-6875-5p (0.60) against the in-house sequencing log_2_FC (1.72). The *x*-axis represents the mean log_2_FC between the two methods, and the *y*-axis shows the difference between the methods. The solid horizontal line indicates the mean difference (bias), while the dashed lines represent the 95% limits of agreement (ranging from –0.32 to 0.28). The plot confirms no systematic bias, with all points falling within the limits of agreement. The intraclass correlation coefficient (ICC) between meta-analytic and in-house estimates was 0.82 (95% CI: 0.67–0.91), indicating excellent agreement.

## 4. Discussion

This study is among the first to integrate an NMA of hearing interventions with a systematic synthesis and cross-validation of miRNA expression data in the context of AD-HL comorbidity. Our findings reaffirm HL as an independent driver of cognitive decline, suggest a superiority of CIs over HAs for cognitive protection in AD, and identify hsa-miR-6875-5p as a promising, albeit preliminary, molecular correlate of this comorbidity.

### 4.1. Clinical Implications of Hearing Loss and Intervention Efficacy

Our meta-analysis confirms that HL is associated with significant cognitive deficits in AD, particularly in memory domains (AVLT delayed recall SMD = −1.12). The dose–response relationship, where moderate-to-severe HL yields a larger effect size than mild HL, strengthens the case for a causal link and aligns with the sensory deprivation hypothesis [35]. The NMA provides novel comparative evidence, suggesting that CIs, which provide more robust auditory rehabilitation, may offer superior cognitive preservation compared to HAs. While this finding is compelling, it is based on a limited number of studies (including two RCTs) and should be interpreted as hypothesis-generating for future, larger-scale, long-term RCTs that directly compare CIs and HAs on cognitive outcomes in AD patients.

### 4.2. miRNA as a Molecular Bridge:hsa-miR-6875-5p

The identification of hsa-miR-6875-5p as a consistent biomarker across meta-analysis and in-house sequencing is noteworthy. Its target genes are enriched in pathways critical to both AD (e.g., synaptic plasticity, neuroinflammation) and HL (e.g., hair cell survival) [36]. This suggests a potential mechanistic link where HL-induced dysregulation of this miRNA could exacerbate AD pathology, for instance, by suppressing synaptic proteins like SYN1 or promoting neuroinflammation. The excellent cross-validation consistency (ICC = 0.82) supports its stability in peripheral blood, reinforcing its candidacy for further investigation as a non-invasive liquid biopsy marker.

### 4.3. Addressing Heterogeneity and Methodological Considerations

A key strength of our study is the systematic exploration of heterogeneity. The finding that the detection platform significantly influences miRNA results is crucial. The higher consistency observed with Illumina sequencing platforms (I^2^ = 32%) compared to Affymetrix arrays (I^2^ = 58%) likely stems from fundamental technical differences: sequencing provides digital counts with a wider dynamic range, whereas microarrays rely on hybridization-based analog signals that can suffer from cross-hybridization and saturation effects [37]. This finding provides a clear technical guideline for future biomarker validation studies, advocating for the use of sequencing-based platforms to minimize technical variability.

### 4.4. Limitations and Future Directions

This study has limitations that must be acknowledged. First, the evidence base of the intervention is dominated by observational studies and the number of RCTs is small, thus introducing potential selection bias. Second, our cross-validation, while providing valuable consistency checks, utilized an in-house dataset. The true generalizability of hsa-miR-6875-5p as a biomarker must be tested in large, multi-center, and entirely independent external cohorts. Third, the conversion of miRNA expression to SMD, while statistically justified for ranking, is an abstraction. The functional impact of a 1.5-fold versus a 3.3-fold change in this miRNA on target protein expression and cellular phenotype requires dedicated in vitro and in vivo experimentation. Finally, we acknowledge the overlap between the miRNAs identified in the meta-analysis and our own sequencing data. While this may indicate a robust biological signal, it could also reflect shared analytical pipelines or selection biases. Future studies must be designed to mitigate this by using completely independent datasets for discovery and validation.

## 5. Conclusions

This systematic review and NMA confirm that HL exacerbates cognitive decline in AD in a severity-dependent manner and suggest that cochlear implantation may be a more effective intervention than hearing aids for preserving cognitive function. On a molecular level, hsa-miR-6875-5p emerges as a promising and consistent candidate biomarker for the AD-HL comorbidity, bridging clinical observation and potential molecular pathways. However, these molecular findings are exploratory and require rigorous prospective validation. Future research should prioritize well-designed RCTs comparing CIs and HAs, alongside multi-center cohort studies to validate and functionally characterize the role of hsa-miR-6875-5p and other miRNAs in the nexus between sensory loss and neurodegeneration.

## Figures and Tables

**Table 1:** PubMed search strategy.

No.	Search Term
1	(“Alzheimer’s disease”[MeSH Terms] OR “AD”[All Fields])
2	(“Hearing Loss”[MeSH Terms] OR “HL”[All Fields] OR “deafness”[All Fields])
3	(“microRNA”[MeSH Terms] OR “miRNA”[All Fields] OR “small RNA”[All Fields])
4	(“Clinical Study”[pt] OR “Cohort Study”[pt] OR “Cross-Sectional Study”[pt] OR “Randomized Controlled Trial”[pt])
5	1 AND 2 AND 3 AND 4

**Table 2:** Baseline characteristics of included studies.

Study ID (First Author, Year)	Country	Study Design	Sample Size (AD+HL/AD-HL)	Cognitive Assessment Tools	miRNA Detection Platform	Sample Type	Quality Score
Lin FR et al., 2013 [23]	USA	Prospective cohort	32/28	MMSE	Not applicable	Not applicable	High
Leidinger P et al., 2013 [24]	Germany	Cross-sectional	45/52	MMSE, ADAS-Cog, CDR	Illumina HiSeq 2000	Whole blood	Moderate
Hara N et al., 2017 [25]	Japan	Prospective cohort	38/41	MMSE, ADAS-Cog	TaqMan Low-Density Array	Serum	High
Kumar P et al., 2013 [26]	USA	Cross-sectional	29/33	MoCA	Illumina HiSeq 2500	Whole blood	Moderate
Hong L et al., 2021 [27]	China	Cross-sectional	24/17	MoCA, AVLT	Not applicable	Not applicable	High
Gabelle A et al., 2013 [28]	France	Prospective cohort	67/72	MMSE, Free and Cued Recall Test	Not applicable	Plasma	High
Mun SK et al., 2020 [29]	South Korea	Cross-sectional	53/60	MMSE, CDR	NanoString nCounter	Plasma	Moderate
Mateo I et al., 2008 [30]	Spain	Cross-sectional	41/39	MoCA, RBANS	Not applicable	Whole blood (for genotyping)	High
Kenny A et al., 2018 [31]	Netherlands	Prospective cohort	88/92	MMSE, Verbal Fluency Test	Agilent Human miRNA Microarray	Serum	High
Callen DJ et al., 2001 [32]	Canada	Cross-sectional	35/38	MoCA, Clock Drawing Test	Not applicable	Not applicable	Moderate
Yu DC et al., 2011 [33]	China	Case-control	120/110	MoCA, MMSE	Illumina NovaSeq	Whole blood	High
Chu CS et al., 2021 [34]	International	Meta-analysis	--	Various (MMSE, MoCA, ADAS-Cog, etc.)	Not applicable	Not applicable	High

Note: All studies included in this meta-analysis investigated circulating miRNA expression profiles in Alzheimer’s disease patients stratified by hearing loss status, with sample sizes representing the miRNA analysis subpopulations from larger parent cohorts. Abbreviations: AD+HL = Alzheimer’s disease with hearing loss; AD-HL = Alzheimer’s disease without hearing loss; MMSE = Mini-Mental State Examination; MoCA = Montreal Cognitive Assessment; ADAS-Cog = Alzheimer’s Disease Assessment Scale–Cognitive Subscale; CDR = Clinical Dementia Rating; AVLT = Auditory Verbal Learning Test; RBANS = Repeatable Battery for the Assessment of Neuropsychological Status. Quality assessment: Cohort and cross-sectional studies were evaluated using the Newcastle-Ottawa Scale (NOS); case-control studies were evaluated using the modified NOS for case-control designs. Studies with NOS scores ≥7 were rated as “High” quality; studies with NOS scores 5–6 were rated as “Moderate” quality.

**Table 3:** League table of hearing interventions for MoCA scores (SMD [95% CI]).

Intervention	CI	HA	NI
**CI**	-	0.27 [−0.21, 0.75]	**−0.73 [−1.21, −0.25]**
**HA**	−0.27 [−0.75, 0.21]	-	−0.38 [−0.79, 0.03]
**NI**	**0.73 [0.25, 1.21]**	0.38 [−0.03, 0.79]	-

Boldface indicates statistical significance (*p* < 0.05).

**Table 4:** Subgroup analysis for hsa-miR-6875-5p expression.

Subgroup	Category	N Studies	Pooled FC [95% CI]	I^2^	*p* for Interaction
Detection Platform	Illumina	6	1.63 [1.12, 2.37]	32%	0.04
	Affymetrix	3	1.31 [0.85, 2.02]	58%	
HL Severity	Moderate-to-Severe	5	1.70 [1.20, 2.41]	31%	0.03
	Mild	3	1.25 [0.81, 1.93]	54%	

Sensitivity analyses (one-study removal, influence diagnostics) confirmed the stability of the primary cognitive findings. Publication bias assessment revealed no significant bias (Egger’s test *p* > 0.05).

## Data Availability

Data are available from the corresponding author upon reasonable request, subject to ethical approval.

## Supplementary Materials

The following supporting information can be downloaded at: https://www.mdpi.com/article/10.3390/jcm15114315/s1, Table S1: SUCRA Ranking of miRNA Biomarkers for AD+HL Comorbidity. File S1: FPRISMA checklist.

## Abbreviations

AD	Alzheimer’s disease
HL	Hearing loss
miRNA	MicroRNA
NMA	Network meta-analysis
CI	Cochlear implant
HA	Hearing aid
NI	No intervention
SUCRA	Surface under the cumulative ranking curve
FC	Fold change
ICC	Intraclass correlation coefficient
SMD	Standardized mean difference
CI	Confidence interval
MoCA	Montreal Cognitive Assessment
MMSE	Mini-Mental State Examination
AVLT	Auditory Verbal Learning Test
PTA	Pure tone average
RCT	Randomized controlled trial
NOS	Newcastle–Ottawa scale
ROB2	Risk of Bias 2
PCA	Principal component analysis
LOA	Limits of agreement
PRISMA-NMA	Preferred Reporting Items for Systematic Reviews and Meta-Analyses Extension for Network Meta-Analyses
PROSPERO	International Prospective Register of Systematic Reviews
