# Supplementary material for: Network Meta-Analysis of Cognitive Impairment and miRNA Expression in Alzheimer’s Disease Patients with Hearing Loss: A Systematic Review and Cross-Validation
Source: J Clin Med. 2026 Jun 3;15(11):4315. doi: 10.3390/jcm15114315 (PMC13257792; doi:10.3390/jcm15114315)
Supplement: Supplementary file 1 [file jcm-15-04315-s001.zip › File S1 PRISMA checklist.pdf]

PRISMA 2020 Checklist

Section and Topic	Item #	Checklist item	Location where item is reported
**TITLE**			
Title	1	Identify the report as a systematic review.	Network Meta-Analysis of Cognitive Impairment and miRNA Expression in Alzheimer's Disease Patients with Hearing Loss: A Systematic Review and Cross-Validation
**ABSTRACT**			
Abstract	2	See the PRISMA 2020 for Abstracts checklist.	Abstract, Page 1
**INTRODUCTION**			
Rationale	3	Describe the rationale for the review in the context of existing knowledge.	Introduction ("Global Burden of Alzheimer's Disease and the Clinical Link with Hearing Loss"; "MicroRNAs as Key Molecular Regulators in AD-HL Comorbidity"; "Limitations of Current Evidence and Rationale for Network Meta-Analysis")

PRISMA 2020 Checklist

Section and Topic	Item #	Checklist item	Location where item is reported
Objectives	4	Provide an explicit statement of the objective(s) or question(s) the review addresses.	Introduction ("Objectives and Innovations of the Present Study")
**METHODS**			
Eligibility criteria	5	Specify the inclusion and exclusion criteria for the review and how studies were grouped for the syntheses.	Materials and Methods ("Eligibility Criteria")
Information sources	6	Specify all databases, registers, websites, organisations, reference lists and other sources searched or consulted to identify studies. Specify the date when each source was last searched or consulted.	Materials and Methods ("Literature Search Strategy - A. Databases and Search Terms")
Search strategy	7	Present the full search strategies for all databases, registers and websites, including any filters and limits used.	Materials and Methods ("Literature Search Strategy"); Table 1
Selection process	8	Specify the methods used to decide whether a study met the inclusion criteria of the review, including how many reviewers screened each record and each report retrieved, whether they worked independently, and if applicable, details of automation tools used in the process.	Materials and Methods ("Eligibility Criteria - C. Screening Process")
Data collection process	9	Specify the methods used to collect data from reports, including how many reviewers collected data from each report, whether they worked independently, any processes for obtaining or confirming data from study investigators, and if applicable, details of automation tools used in the process.	Materials and Methods ("Data Extraction and Quality Assessment - A. Data Extraction")
Data items	10a	List and define all outcomes for which data were sought. Specify whether all results that were compatible with each outcome domain in each study were sought (e.g. for all measures, time points, analyses), and if not, the methods used to decide which results to collect.	Materials and Methods ("Eligibility Criteria"; "Data Extraction and Quality Assessment - A. Data Extraction")

PRISMA 2020 Checklist

Section and Topic	Item #	Checklist item	Location where item is reported
	10b	List and define all other variables for which data were sought (e.g. participant and intervention characteristics, funding sources). Describe any assumptions made about any missing or unclear information.	Materials and Methods ("Data Extraction and Quality Assessment - A. Data Extraction")
Study risk of bias assessment	11	Specify the methods used to assess risk of bias in the included studies, including details of the tool(s) used, how many reviewers assessed each study and whether they worked independently, and if applicable, details of automation tools used in the process.	Materials and Methods ("Data Extraction and Quality Assessment - B. Quality Assessment")
Effect measures	12	Specify for each outcome the effect measure(s) (e.g. risk ratio, mean difference) used in the synthesis or presentation of results.	Materials and Methods ("Statistical Analysis - A. Traditional Meta-Analysis")
Synthesis methods	13a	Describe the processes used to decide which studies were eligible for each synthesis (e.g. tabulating the study intervention characteristics and comparing against the planned groups for each synthesis (item #5)).	Materials and Methods ("Eligibility Criteria"; "Statistical Analysis - B. Network Meta-Analysis")
	13b	Describe any methods required to prepare the data for presentation or synthesis, such as handling of missing summary statistics, or data conversions.	Materials and Methods ("Statistical Analysis - A. Traditional Meta-Analysis")
	13c	Describe any methods used to tabulate or visually display results of individual studies and syntheses.	Materials and Methods ("Statistical Analysis - B. Network Meta-Analysis -

Section and Topic	Item #	Checklist item	Location where item is reported
			Outcome Analysis and Visualization")
	13d	Describe any methods used to synthesize results and provide a rationale for the choice(s). If meta-analysis was performed, describe the model(s), method(s) to identify the presence and extent of statistical heterogeneity, and software package(s) used.	Materials and Methods ("Statistical Analysis - A. Traditional Meta-Analysis"; "B. Network Meta-Analysis")
	13e	Describe any methods used to explore possible causes of heterogeneity among study results (e.g. subgroup analysis, meta-regression).	Materials and Methods ("Statistical Analysis - C. Subgroup Analysis and Meta-Regression")
	13f	Describe any sensitivity analyses conducted to assess robustness of the synthesized results.	Materials and Methods ("Statistical Analysis - D. Publication Bias and Sensitivity Analysis")
Reporting bias assessment	14	Describe any methods used to assess risk of bias due to missing results in a synthesis (arising from reporting biases).	Materials and Methods ("Statistical Analysis - D. Publication Bias and Sensitivity Analysis")
Certainty assessment	15	Describe any methods used to assess certainty (or confidence) in the body of evidence for an outcome.	Not reported (no certainty/GRADE assessment described)
**RESULTS**			
Study selection	16a	Describe the results of the search and selection process, from the number of records identified in the search to the number of studies included in the review, ideally using a flow diagram.	Results ("Literature Screening and

Section and Topic	Item #	Checklist item	Location where item is reported
			Study Characteristics - A. PRISMA-Compliant Literature Screening"); Figure 4
	16b	Cite studies that might appear to meet the inclusion criteria, but which were excluded, and explain why they were excluded.	Partly reported in Results ("Literature Screening and Study Characteristics - A. PRISMA-Compliant Literature Screening"); reasons for exclusion are summarized, but excluded studies are not cited individually
Study characteristics	17	Cite each included study and present its characteristics.	Results ("Literature Screening and Study Characteristics - B. Baseline Characteristics of Included Studies"); Table 4
Risk of bias in studies	18	Present assessments of risk of bias for each included study.	Materials and Methods ("Data Extraction and Quality Assessment - B. Quality Assessment"); Results ("Baseline Characteristics of Included Studies"); Table 2; Table 4

Section and Topic	Item #	Checklist item	Location where item is reported
Results of individual studies	19	For all outcomes, present, for each study: (a) summary statistics for each group (where appropriate) and (b) an effect estimate and its precision (e.g. confidence/credible interval), ideally using structured tables or plots.	Results ("Traditional Meta-Analysis Results"); Figure 5A; Figure 5B; Figure 6; Table 5
Results of syntheses	20a	For each synthesis, briefly summarise the characteristics and risk of bias among contributing studies.	Results ("Literature Screening and Study Characteristics - B. Baseline Characteristics of Included Studies"); quality summary in text and Table 4
	20b	Present results of all statistical syntheses conducted. If meta-analysis was done, present for each the summary estimate and its precision (e.g. confidence/credible interval) and measures of statistical heterogeneity. If comparing groups, describe the direction of the effect.	Results ("Traditional Meta-Analysis Results"; "Network Meta-Analysis Results"); Figure 5A; Figure 5B; Figure 6; Figure 7; Table 5
	20c	Present results of all investigations of possible causes of heterogeneity among study results.	Results ("Heterogeneity, Publication Bias, and Sensitivity Analysis - A. Heterogeneity Sources"); Table 6
	20d	Present results of all sensitivity analyses conducted to assess the robustness of the synthesized results.	Results ("Heterogeneity, Publication Bias, and Sensitivity Analysis - C. Sensitivity Analysis")

PRISMA 2020 Checklist

Section and Topic	Item #	Checklist item	Location where item is reported
Reporting biases	21	Present assessments of risk of bias due to missing results (arising from reporting biases) for each synthesis assessed.	Results ("Heterogeneity, Publication Bias, and Sensitivity Analysis - B. Publication Bias"); Figure 8
Certainty of evidence	22	Present assessments of certainty (or confidence) in the body of evidence for each outcome assessed.	Not reported (certainty of evidence not assessed)
**DISCUSSION**			
Discussion	23a	Provide a general interpretation of the results in the context of other evidence.	Discussion ("Core Findings and Clinical Implications"; "Clinical Correlation Between Hearing Loss and Cognitive Impairment"; "miRNA as Molecular Bridges: From Biomarkers to Mechanisms")
	23b	Discuss any limitations of the evidence included in the review.	Discussion ("Limitations and Future Directions - Study Limitations")
	23c	Discuss any limitations of the review processes used.	Partly reported in Discussion ("Limitations and Future Directions"); limitations of the review process are not discussed separately

PRISMA 2020 Checklist

Section and Topic	Item #	Checklist item	Location where item is reported
	23d	Discuss implications of the results for practice, policy, and future research.	Discussion ("Limitations and Future Directions - Translational and Research Priorities"); "5. Conclusions"; "Clinical and Research Implications"
**OTHER INFORMATION**			
Registration and protocol	24a	Provide registration information for the review, including register name and registration number, or state that the review was not registered.	Materials and Methods ("Study Design and Registration")
	24b	Indicate where the review protocol can be accessed, or state that a protocol was not prepared.	Not reported (protocol registration is stated, but protocol access location is not provided)
	24c	Describe and explain any amendments to information provided at registration or in the protocol.	Not reported clearly (registration number is mentioned as updated before manuscript submission, but amendments are not described)
Support	25	Describe sources of financial or non-financial support for the review, and the role of the funders or sponsors in the review.	Funding; Acknowledgments; Conflicts of Interest
Competing interests	26	Declare any competing interests of review authors.	Conflicts of Interest

PRISMA 2020 Checklist

Section and Topic	Item #	Checklist item	Location where item is reported
Availability of data, code and other materials	27	Report which of the following are publicly available and where they can be found: template data collection forms; data extracted from included studies; data used for all analyses; analytic code; any other materials used in the review.	Data Availability Statement; Supplementary Materials

From: Page MJ, McKenzie JE, Bossuyt PM, Boutron I, Hoffmann TC, Mulrow CD, et al. PRISMA 2020 explanation and elaboration: Updated guidance and exemplars for reporting systematic reviews. BMJ 2021;372:n160. doi: 10.1136/bmj.n160. This work is licensed under CC BY 4.0. To view a copy of this license, visit https://creativecommons.org/licenses/by/4.0/
