# Supplementary material for: Network Meta-Analysis of Cognitive Impairment and miRNA Expression in Alzheimer’s Disease Patients with Hearing Loss: A Systematic Review and Cross-Validation
Source: J Clin Med. 2026 Jun 3;15(11):4315. doi: 10.3390/jcm15114315 (PMC13257792; doi:10.3390/jcm15114315)
Supplement: Supplementary file 1 [file jcm-15-04315-s001.zip › Table S1 SUCRA Ranking of miRNA Biomarkers for AD+HL Comorbidity. .pdf]

Table S1. SUCRA Ranking of miRNA Biomarkers for AD+HL Comorbidity

Rank	miRNA Name	SUCRA Score	Notes
1	PC-5p-14597_152	0.91	Strongest and most consistent association with AD+HL
2	hsa-miR-6875-5p	0.85	Dense network connectivity with cognitive outcome measures
3	hsa-miR-4435	0.72	Moderate association with AD+HL
4	hsa-miR-1234-3p_R-1	0.28	Weakest association among the ranked miRNAs
